# Disability and its correlates among older adults with common mental disorders in China: a 5-year follow-up study

**DOI:** 10.3389/fpsyt.2026.1907148

**Published:** 2026-10-07

**Authors:** Shuang Liu, Xiaofei Hou, Huifang Yin, Yueqin Huang, Minghui Li, Zhengjing Huang, Mi Hu, Shulin Chen, Changgui Kou, Yongping Yan, Hua Ding, Jie Yan, Jing Wen, Cunxian Jia, Qiang Li, Zhaorui Liu, Guangming Xu

**Affiliations:** 1 Tianjin Anding Hospital, Mental Health Center, Tianjin Medical University, Tianjin, China; 2 Peking University Sixth Hospital, Peking University Institute of Mental Health, NHC Key Laboratory of Mental Health (Peking University), National Clinical Research Center for Mental Disorders (Peking University Sixth Hospital), Beijing, China; 3 National Center for Chronic and Noncommunicable Disease Control and Prevention, Beijing, China; 4 Xiangya School of Public Health, Changsha, China; 5 Department of Psychological and Behavior Science, Zhejiang University, Hangzhou, China; 6 Department of Epidemiology and Biostatistics, School of Public Health, Jilin University, Changchun, China; 7 Department of Epidemiology, The Fourth Military Medical University, Xi’an, China; 8 Institute of Social Science Survey, Peking University, Beijing, China; 9 School of Government, Peking University, Beijing, China; 10 Department of Epidemiology and Health Statistics, School of Public Health and Management at Ningxia Medical University, Yinchuan, China; 11 Department of Epidemiology, School of Public Health, Cheeloo College of Medicine, Shandong University, Jinan, China

**Keywords:** common mental disorders, correlates, disability, follow-up study, older adults

## Abstract

**Background:**

Common mental disorders (CMDs) represent a category of conditions characterized by a high rate of disability and a significant social burden. This study aims to describe disability at baseline and five years later among older adults with 12-month CMDs and to investigate its correlates.

**Methods:**

Based on the nationally representative China Mental Health Survey (CMHS), a five-year follow-up cohort study was conducted, in which older adults with 12-month common mental disorders (CMDs) at baseline were reassessed. Follow-up assessments were conducted by telephone, and disability was measured using the World Health Organization Disability Assessment Schedule 2.0. Disability prevalence at baseline and five-year follow-up and its correlates were analysed.

**Results:**

The baseline analyses included 582 older adults with CMDs, of whom 134 completed the five-year follow-up and were included in the follow-up analyses. The prevalence of disability at baseline and five years later were 57.1% and 19.3%, respectively. Older age (≥70 years vs. 60–64 years: RR = 1.12, 95% CI: 1.06-1.19), residence in the Western region (vs. Eastern: RR = 1.09, 95% CI: 1.02-1.16), physical comorbidity (1–2 diseases: RR = 1.10, 95% CI: 1.02-1.18; ≥3 diseases: RR = 1.31, 95% CI: 1.22-1.41), comorbidity of multiple CMDs (RR = 1.21, 95% CI: 1.14-1.29), and sleep disturbances (RR = 1.17, 95% CI: 1.10-1.24) were associated with higher disability scores. Higher educational level (junior high school and above vs. literate or below primary school) was associated with lower long-term disability among participants with CMDs (RR = 0.85; 95% CI: 0.74-0.97). Living in urban areas (RR = 0.80; 95% CI: 0.69-0.92) was associated with lower long-term disability among individuals with anxiety disorders.

**Conclusion:**

Long-term disability persists among older adults with CMDs who survived and were reachable despite an overall decline. Modifiable factors, including physical and mental comorbidity and sleep disturbances, were associated with higher baseline disability, whereas higher education and urban living were associated with a reduced risk of long-term disability. Integrated interventions are needed to reduce disability burden.

## 1 Introduction

The aging population poses a substantial burden of mental disorders among older adults, with about 14 percent of individuals aged 60 and older suffering from mental disorders globally (1). Moreover, mental disorders account for 10.6 percent of the overall disabilities observed in this age group (2). In China, the prevalence of mental disorders among older adults was 24.04%, with a disability rate attributable to these disorders of 1.09 percent (3). The China Mental Health Survey (CMHS) conducted in 2013 among individuals aged 65 and older revealed that anxiety disorders (ADs), mood disorders (MDs), and substance use disorders (SUDs) were the top three common mental disorders (CMDs), with respective 12-month weighted prevalence rates of 4.7%, 3.9%, and 0.3% (4).

A multitude of studies have shown that mental disorders increase the risk of disability (5). As age increases, older adults with mental disorders experience a significant worsening of their level of disability, particularly affecting their self-care abilities and capacity to understand and communicate (6). Additionally, a longitudinal study has indicated that mental disorders may be associated with long-term disabilities, including limited mobility, difficulties in performing tasks independently, and challenges in communicating with others (7). Mental disorders significantly affect the levels of disability and overall quality of life in older adults compared to most chronic physical illnesses (8).

Few studies have investigated the factors correlated with disability among older adults with CMDs. One study showed that some factors related to disability among Chinese older adults include demographic factors, health behaviors, social support (e.g., spouse, family income), smoking, chronic disease, and mental resilience (9). Furthermore, the stigma linked to mental disorders often leads individuals to seek help, treatment, or social support rarely (5). Along with unhealthy eating patterns, diminished physical activity, and lower socioeconomic status, older adults with mental disorders may face an increased risk of long-term disability (10, 11). Previous studies have focused on disability resulting from specific mental disorder or symptoms rather than CMDs among older adults (12–14). Few studies have distinguished between baseline and long-term disability after years of follow-up, or examined their distinct correlates, among Chinese adults (15, 16), and none have done so specifically in older adult populations.

Therefore, there is an urgent need to provide valuable information about long-term disabilities associated with CMDs and related factors to prevent older adults with CMDs from suffering from disabilities. Thus, to explore the trends of long-term disability among older adults with mental disorders, a five-year follow-up study was conducted after the completion of the CMHS. This research defines long-term disability as functional disability observed five years later, utilizing the World Health Organization Disability Assessment Schedule 2.0 (WHODAS-2.0). This study aimed to: 1) describe the prevalence of disability at baseline and five-year follow-up among older adults with CMDs; and 2) investigate the correlating factors with disability both at baseline and five years later.

## 2 Methods

### 2.1 Sample and procedures

#### 2.1.1 Sample

The baseline sample was obtained from the CMHS, a nationwide epidemiological study on mental health conditions and services in China conducted between 2012 and 2015. Built upon the national Disease Surveillance Point (DSP) system, the CMHS deployed a multi-stage, disproportionate stratified sampling strategy. By covering all 31 provincial-level administrative units across China, this design enabled the recruitment of a nationally representative sample. The survey sampled 40964 households at 157 nationwide sites, which were distributed across 628 sub-districts and townships and included 1256 urban communities and rural villages. Valid diagnostic interviews were obtained for 28140 adults aged 18 years or older. Detailed descriptions of the study design and specific methodological aspects of the CMHS can be found in previously published articles (4, 17, 18).

The five-year follow-up was conducted from April to June 2018 (15). All participants who completed baseline diagnostic interviews in the CMHS were eligible for follow-up. Participants were excluded if (1): repeated contact attempts failed to establish telephone connection (2); participants declined to take part in the follow-up interview (3); participants could not complete the interview independently owing to severe physical or mental disorders, relocation without available telephone contact, while no qualified proxy informant was accessible to complete the interview.

Among the 28140 participants at baseline, 7454 were aged 60 years or older. The present study included those aged 60 years or older who met diagnostic criteria for 12-month CMDs at baseline in the CMHS, with all eligible followed participants included in the follow-up analyses (**Figure 1**). For participants with successful follow-up assessment, the individual follow-up interval varied owing to staggered baseline data collection across 2012-2015. Among the 134 re-interviewed participants, the follow-up duration was 4.47 years (median; IQR 4.41–4.56; range 4.31–4.69). This study refers to the prespecified target time-window as the “5-year follow-up”.

**Figure 1:**
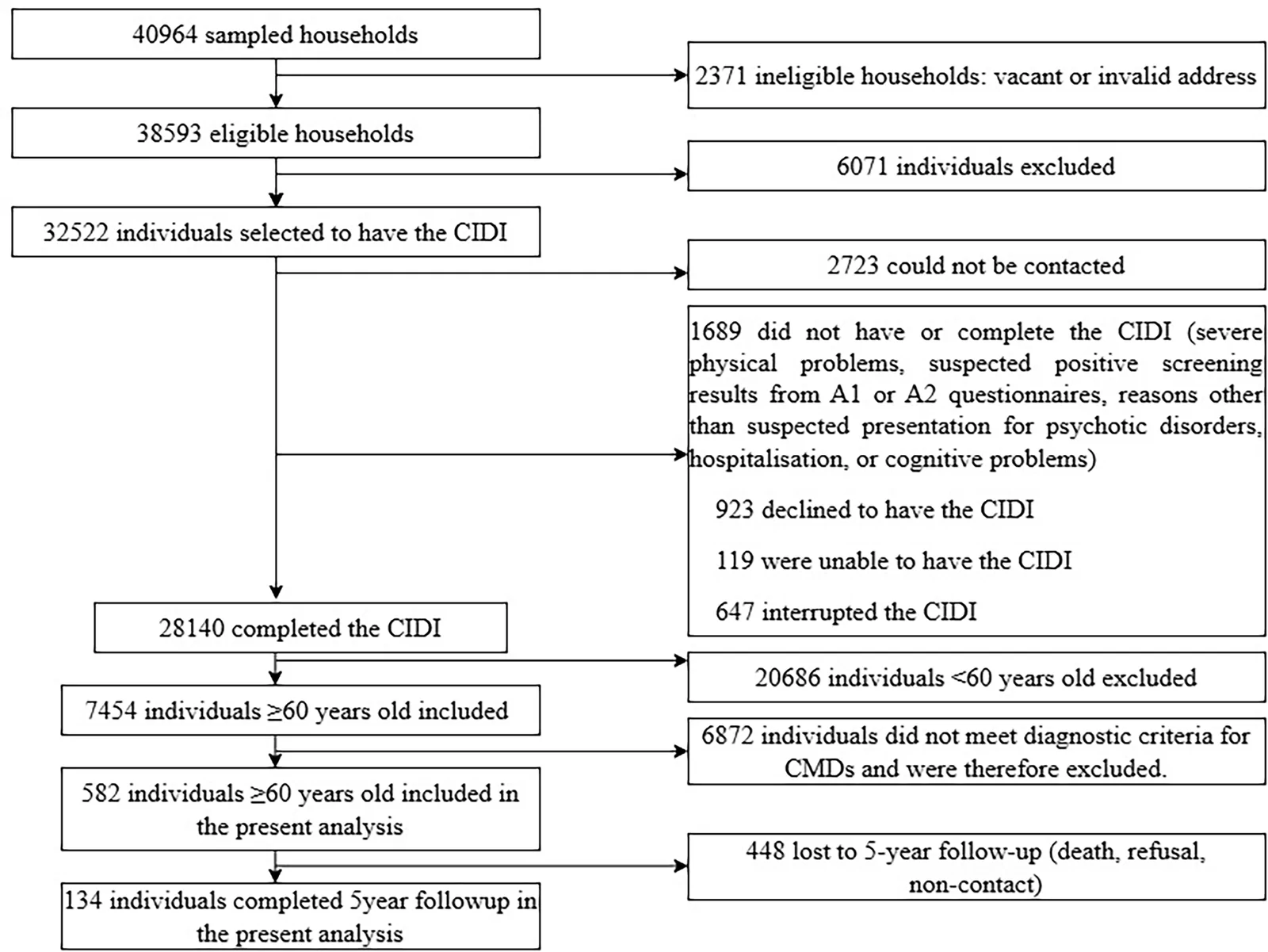
Participant flow diagram. CIDI, Composite International Diagnostic Interview; CMDs, Common mental disorders.

#### 2.1.2 Procedures

Participants were interviewed at baseline by trained interviewers using the Chinese computer-assisted personal interview (CAPI) version of the Composite International Diagnostic Interview 3.0 (CIDI-3.0). The follow-up was conducted using the computer-assisted telephone interview (CATI) method. Before formally commencing participation in the follow-up study, 50 interviewers completed a 2-day training course covering the use of CATI, study procedures, skills for conducting telephone interviews, the content of the questionnaire, and methods for ensuring quality control, culminating in the successful completion of a final examination. Additionally, four supervisors were assigned to review the interview criteria. Participants who met the follow-up standards were interviewed, and proxy interviews conducted by the informants were permitted. For specific details regarding the allowance of proxy interviews by informants, please refer to the published article (15).

All baseline information, including WHODAS-2.0 score, 12-month CMD diagnoses, CMD comorbidity, physical health conditions, sleep disturbance, household income, and other demographic and clinical covariates, was exclusively obtained from the baseline 2012–2015 CMHS. At the 2018 follow-up, only the WHODAS-2.0 disability outcome was reassessed. No repeat diagnostic interviews or re-measurements of physical conditions, sleep status, or socioeconomic covariates were conducted.

The studies involving humans were approved by the Ethics Committees of Tianjin Anding Hospital and the Sixth Hospital of Peking University. The studies were conducted in accordance with the local legislation and institutional requirements. Written informed consent was obtained in the baseline survey in accordance with the approved protocol. For the five-year telephone follow-up, oral informed consent was obtained from participants and informants via telephone, and the consent process was audio-recorded in accordance with the ethics committee-approved procedures. Detailed descriptions of the specific quality controls have been published elsewhere (15).

### 2.2 Measurement

#### 2.2.1 Diagnosis of common mental disorders

Mental disorders were diagnosed through the CIDI-3.0, a comprehensive structured interview tool conducted by trained lay interviewers (19). The CIDI-3.0 has demonstrated satisfactory validity and has been employed in China (4) as well as several other nations for epidemiological research (20, 21).

The diagnosis of CMDs over 12 months was established based on the criteria and definitions specified in the Diagnostic and Statistical Manual, Fourth Edition (DSM-IV) (18), which is divided into three categories (1): ADs, including social phobia (SO), panic disorder (PD), obsessive-compulsive disorder (OCD), specific phobia (SP), Agoraphobia with/out panic disorder (AGP), generalized anxiety disorder(GAD), and substance-induced anxiety disorder (2); MDs, comprising depressive disorder (DEP), depressive disorder not otherwise specified, bipolar disorder (BD), BD I, BD II, BD not otherwise specified, dysthymic disorder (DD), major depressive disorder (MDD), and substance-induced mood disorder; and (3) SUDs, including drug use disorder (DUD) and alcohol use disorder (AUD).

#### 2.2.2 Assessment for disability

The functional disability of the participants was assessed at baseline and after five years using the WHODAS-2.0 (22). The Chinese version of WHODAS-2.0 has shown robust reliability and validity (23). Functional disability within 30 days preceding the assessment was evaluated across several domains: 1) Understanding and communication (cognitive domain); 2) Moving and getting around (mobility domain); 3) Personal hygiene, dressing, eating, and the ability to live independently (self-care domain); 4) Engaging with other individuals (social interaction domain); 5) Difficulties in carrying out work or normal activities (life activities domain); 6) Participation in community activities and societal engagement (participation domain). Total WHODAS-2.0 scores were computed following the official WHO scoring algorithms, with scores from the six functional domains aggregated to reflect overall disability severity. Unified computation rules were applied to ensure consistency between baseline and follow-up data.

We dichotomized the score to define the presence of “substantial disability” primarily to allow for a more intuitive and interpretable estimate of disability prevalence. The 90th percentile threshold was selected following the method used in World Mental Health Surveys (16, 24, 25). This threshold was calculated based on the WHODAS-2.0 score distribution among all adults aged in the nationally representative baseline CMHS sample, corresponding to a cut-off score of 52. This unified cut-off was applied identically to both baseline and five-year follow-up assessments.

### 2.3 Potential predictors

At baseline, data on potential predictors were collected, encompassing seven demographic factors: age (60-64, 65-69, and 70 years and older), educational level (junior high school and above, primary school, literate or below primary school), marital status (married/cohabitating, separated/widowed/divorced/never married), area of residence (rural or urban), annual household income categories (<10,500 Yuan, 10500 -42499 Yuan, and≥42,500 Yuan), sex (female or male), and region (eastern, central, western).

Physical diseases and sleep disturbances over 12 months were assessed using the CIDI-3.0. This assessment included sleep disturbances that persisted for two weeks or more within the past year. Data on physical diseases were collected through self-reports and encompassed conditions such as asthma, heart disease, diabetes, tuberculosis, stomach or intestinal ulcers, high blood pressure, rheumatic fever or arthritis, stroke, chronic lung disease, and chronic headaches. The self-reported data on physical diseases were categorized into three groups: individuals with no physical diseases, those with one or two physical diseases, and those suffering from three or more physical diseases.

Comorbidity of CMDs was assessed via the CIDI-3.0 and defined as the presence of disorders from two or more major diagnostic categories (MDs, ADs, SUDs). Co-occurrence of different subtypes within the same major category was not counted as comorbidity.

### 2.4 Contact information

Contact information of the participants was collected for monitoring purposes at baseline and five years later. This information included their addresses, intentions to relocate, and the contact details of contributors and informants.

### 2.5 Statistical analysis

Analytical weights for the baseline CMHS can be found elsewhere published (15, 16), combining three sequential components: sampling-design weights, multi-level non-response adjustment weights, and post-stratification weights. The five-year follow-up weights were constructed following the framework of the CMHS. The main distinction from baseline weights was that non-response weights for the follow-up cohort were additionally adjusted according to the diagnosis of mental disorder. This comprehensive weighting approach accounts for the complex sampling design, differential non-response, and population representativeness, thereby reducing potential selection bias arising from the high attrition rate.

The structured prompting procedures of CAPI at baseline and CATI at follow-up helped minimise item-level missing responses. For missing WHODAS-2.0 items, participants with valid responses to at least half of the items within a domain were retained, with missing values imputed using domain-specific mean scores. If more than half of the items in any domain were missing, the total score was treated as missing and the participant was excluded from relevant analyses.

To assess potential selection bias due to the high attrition rate, baseline characteristics were compared between participants who completed the five-year follow-up and those who were lost to follow-up. Categorical variables were presented as frequencies with proportions and compared using chi-square tests. Continuous variables were presented as medians with interquartile ranges (IQRs) and compared using the Wilcoxon rank-sum test due to the skewed distribution. A multivariable logistic regression model was additionally constructed to identify independent baseline predictors of attrition.

Descriptive analyses used frequencies to characterise the sample and the prevalence of substantial disability, defined using the 90th percentile cut-off of the WHODAS-2.0 global score. For participants with complete baseline and five-year follow-up data, McNemar’s test was used to assess changes in binary substantial disability status. Weighted prevalence estimates are reported with 95% confidence intervals (CIs), calculated using the Taylor series linearisation method to account for sampling weights and clustering. In the regression models, the continuous WHODAS-2.0 total score was treated as the dependent variable, with results reported as relative risks (RRs) and 95% CIs. Given the skewed distribution and overdispersion, negative binomial regression was employed to identify correlates of WHODAS-2.0 total score at baseline and follow-up rather than ordinary linear regression (26, 27). Negative binomial provided the best fit and was therefore preferred over log-transformed linear and zero-inflated negative binomial models. Owing to limited sample sizes at follow-up, the full set of exploratory analyses, including three models (Model 1: baseline WHODAS-adjusted univariate; Model 2: demographic correlates multivariable; Model 3: demographic and clinical correlates multivariable), was presented in the Supplementary Materials. All subgroup analyses were considered exploratory, and multivariable regression was not performed for SUDs due to insufficient sample size.

In addition, to assess the robustness of findings under different assumptions about missing data, sensitivity analyses were conducted comparing unweighted complete-case analysis with weighted analysis using the final analytical weights described above.

The significance level of α=0.05 was consistently maintained throughout the analysis. Statistical evaluations were conducted using SAS version 9.4.

## 3 Results

### 3.1 Demographic characteristics of the participants with CMDs at baseline and five years later

The baseline sample comprised 582 individuals aged 60 years or older who met the criteria for 12-month CMDs. Among them, 134 (23.0%) completed the five-year follow-up assessment. Of the 134 valid follow-up participants, 27 (20.2%) were assessed through eligible proxy informants, while 107 (79.8%) provided self-reported information.

As shown in **Supplementary Table S1**, comparisons of baseline characteristics between participants who were followed up and those who were lost to follow-up revealed no significant differences in demographic characteristics, clinical variables, or baseline disability (p>0.05), except for marital status (p=0.040) and CMD diagnosis type (p=0.047). Multivariable logistic regression further showed that no baseline variable was independently associated with loss to follow-up (**Supplementary Table S2**).

At baseline, 43.1% were male, 74.6% were married or cohabitating, and 50.7% had an educational level of literate or below primary school, with an average age of 66.5 ± 5.6 years old. Five years later, among the 134 participants interviewed, 43.3% were male, and 52.2% were aged 60-64, with an average age of 65.6 ± 5.2 years old at baseline. **Table 1** provides detailed information regarding the demographic characteristics of participants aged 60 years and older with CMDs at baseline and five years later.

**Table 1:** Demographic characteristics of the participants with CMDs at baseline and five years later.

Variables		2013(n=582)		2018(n=134)	
Sociodemographic characteristics	Category	Frequency n	Proportion (%)	Frequency n	Proportion (%)
Age	60-64	266	45.7	70	52.2
	65-69	169	29.0	34	25.4
	≥70	147	25.3	30	22.4
Sex	Male	251	43.1	58	43.3
	Female	331	56.9	76	56.7
Educational level	Literate/below primary school	295	50.7	61	45.5
	Primary school	164	28.2	39	29.1
	Junior high school and above	123	21.1	34	25.4
Marital status	Married/Cohabitating	434	74.6	109	81.3
	Separated/Widowed/ Divorced/Never Married	148	25.4	25	18.7
Annual household income	<10500	308	52.9	61	45.5
	10500-42499	164	28.2	42	31.3
	≥42500	110	18.9	31	23.1
Region	Eastern	171	29.4	37	27.6
	Central	235	40.4	61	45.5
	Western	176	30.2	36	26.9
Area of residence	Rural	338	58.1	74	55.2
	Urban	244	41.9	60	44.8

CMDs, Common mental disorders.

### 3.2 The prevalence of disability at baseline and five years later

**Table 2** shows the prevalence of disability at baseline and five years later among older adults with main 12-month CMDs. For several diagnostic subgroups with limited sample size, estimates are reported descriptively only in the Supplementary Materials (**Supplementary Table S3**). The weighted prevalence of disability for any CMDs at baseline and follow-up were 57.1% and 19.3%, respectively. For MDs, the weighted prevalence of disability at baseline and five years later were 70.2% and 28.9%, respectively. Specifically, for MDD, the weighted prevalence of substantial disability was 74.9% at baseline and 25.4% after five years. In the case of DD, the weighted prevalence of disability at baseline and five years later were 86.4% and 20.3%, respectively.

**Table 2:** The weighted prevalence of disability among participants with CMDs at baseline and five years later. .

Common mental disorder category	2013			2018		
	Disability			Disability		
	N	n	Weighted % (95% CI)	N	n	Weighted % (95% CI)
**Mood disorders**	317	200	70.2 (62.1-78.4)	71	25	28.9 (10.3-47.4)
Depressive disorders	293	182	69.9 (60.9-78.8)	65	24	28.6 (8.6-48.7)
Major depressive disorder	237	163	74.9 (64.5-85.3)	57	22	25.4 (7.2-43.6)
Dysthymic disorder	106	76	86.4 (80.7-92.2)	22	9	-^*^
Depressive disorder not otherwise specified	48	15	46.2 (37.4-54.9)	6	2	-^*^
Bipolar disorders	19	13	70.5 (68.0-72.9)	6	1	-^*^
Substance-induced mood disorder	6	6	-^*^	–	–	–
**Anxiety disorders**	315	166	56.4 (46.8-65.9)	86	21	14.9 (3.8-25.9)
Panic disorder	34	22	66.9 (60.9-72.8)	12	4	-^*^
Agoraphobia with/out panic disorder	18	14	87.5 (85.9-88.9)	8	4	-^*^
Specific phobia	164	83	54.6 (43.9-65.4)	44	9	-^*^
Social phobia	27	21	91.8 (87.8-95.9)	8	2	-^*^
Obsessive compulsive disorder	113	62	57.5 (41.9-72.9)	24	5	-^*^
Generalized anxiety disorder	19	11	60.9 (25.2-96.8)	11	4	-^*^
Substance-induced anxiety disorder	14	13	-^*^	5	2	-^*^
**Substance use disorders**	57	20	33.7 (12.9-54.5)	9	2	-^*^
**Any common mental disorder**	582	307	57.1 (49.0-65.1)	134	35	19.3 (9.0-29.7)

CMDs, Common mental disorders; CI, Confidence Interval; ^*^The 95% CI are not reported where the sample was too small to permit reliable estimation; descriptive subtype estimates are provided in **Supplementary Table S3**. Bold values indicate higher weighted prevalence of disability.

For ADs, the weighted prevalence of disability at baseline and five years later were 56.4% and 14.9%, respectively. Regarding PD, the weighted prevalence was 66.9% at baseline and 22.5% after five years. For OCD, the weighted prevalence of disability at baseline and five years later were 57.5% and 27.1%, respectively. For GAD, the weighted prevalence was 60.9% at baseline and 22.1% after five years.

For SUDs, the weighted prevalence of substantial disability was 33.7% at baseline and 28.0% after five years, while the weighted prevalence for AUDs at baseline and five years later was 18.9% and 36.0%, respectively.

Among the 134 participants with paired baseline and follow-up data, weighted baseline substantial disability prevalence was 51.7% (95% CI: 40.9-62.5%), and 19.3% (95% CI: 9.0-29.7%) at 5-year follow-up. The decrease in disability prevalence was statistically significant for CMDs overall, MDs, and ADs (all p<0.001). The McNemar’s test was not performed for SUDs due to insufficient sample size (Detailed in **Supplementary Table S3**).

### 3.3 Correlates of disability severity at baseline

The results of the multivariate negative binomial regression on correlates of baseline WHODAS-2.0 total score are shown in **Table 3**. Among older adults with 12-month CMDs, higher disability scores were significantly associated with older age (≥70 years vs. 60–64 years: RR = 1.12; 95% CI: 1.06-1.19), residing in western regions (vs. Eastern: RR = 1.09; 95% CI: 1.02-1.16), comorbidity of physical diseases (1–2 physical diseases: RR = 1.10; 95% CI: 1.02-1.18; three or more physical diseases: RR = 1.31; 95% CI: 1.22-1.41), comorbidity of CMDs (RR = 1.21; 95% CI: 1.14-1.29) and sleep disturbances (RR = 1.17; 95% CI: 1.10-1.24).

**Table 3:** Correlates of WHODAS 2.0 score at baseline regarding CMDs, MDs, ADs: multivariate negative binomial regression^a^.

Variables	Category	CMDs (n=582)		MDs (n=317)		ADs (n=315)	
		RR (95% CI)	*P*	RR (95% CI)	*P*	RR (95% CI)	*P*
Age	60-64	Ref.	-	Ref.	-	Ref.	-
	65-69	1.01 (0.95-1.07)	0.786	0.92 (0.84-1.01)	0.076	1.05 (0.98-1.14)	0.187
	≥70	1.12 (1.06-1.19)	**<0.001**	1.03 (0.95-1.13)	0.487	1.16 (1.08-1.25)	**<0.001**
Sex	Female	Ref.	-	Ref.	-	Ref.	-
	Male	0.97 (0.92-1.02)	0.196	0.99 (0.92-1.08)	0.896	1.04 (0.98-1.11)	0.221
Marital status	Married/Cohabitating	Ref.	-	Ref.	-	Ref.	-
	Separated/Widowed/ Divorced/Never Married	0.98 (0.93-1.05)	0.596	1.01 (0.93-1.10)	0.754	1.05 (0.98-1.13)	0.192
Educational level	Literate/below primary school	Ref.	-	Ref.	-	Ref.	-
	Primary school	1.03 (0.97-1.10)	0.294	1.10 (1.00-1.20)	**0.039**	0.96 (0.89-1.04)	0.321
	Junior high school and above	0.99 (0.92-1.06)	0.699	1.03 (0.93-1.14)	0.616	0.96 (0.88-1.04)	0.274
Annual household income	<10500	Ref.	-	Ref.	-	Ref.	-
	10500-42499	0.98 (0.92-1.03)	0.436	0.96 (0.89-1.05)	0.374	1.00 (0.94-1.07)	0.941
	≥42500	0.97 (0.90-1.04)	0.340	0.97 (0.87-1.09)	0.644	1.02 (0.94-1.11)	0.587
Region	Eastern	Ref.	-	Ref.	-	Ref.	-
	Central	1.06 (1.00-1.12)	0.070	1.09 (0.99-1.19)	0.074	1.02 (0.95-1.10)	0.537
	Western	1.09 (1.02-1.16)	**0.009**	1.11 (1.01-1.22)	**0.024**	1.06 (0.99-1.15)	0.110
Area of residence	Rural	Ref.	-	Ref.	-	Ref.	-
	Urban	0.97 (0.92-1.02)	0.255	0.98 (0.90-1.06)	0.641	0.96 (0.90-1.02)	0.228
Comorbidity of physical diseases	0	Ref.	-	Ref.	-	Ref.	-
	1-2	1.10 (1.02-1.18)	**0.016**	1.04 (0.92-1.18)	0.492	1.19 (1.08-1.30)	**<0.001**
	≥3	1.31 (1.22-1.41)	**<0.001**	1.23 (1.09-1.39)	**<0.001**	1.35 (1.23-1.48)	**<0.001**
Comorbidity of CMDs	No	Ref.	-	Ref.	-	Ref.	-
	Yes	1.21 (1.14-1.29)	**<0.001**	1.14 (1.06-1.23)	**<0.001**	1.29 (1.21-1.38)	**<0.001**
Sleep disturbances	No	Ref.	-	Ref.	-	Ref.	-
	Yes	1.17 (1.10-1.24)	**<0.001**	1.21 (1.10-1.34)	**<0.001**	1.14 (1.06-1.23)	**<0.001**

WHODAS, world health organization disability assessment schedule; CMDs, Common mental disorders; MDs, Mood disorders; ADs, Anxiety disorders; RR, rate ratio; CI, Confidence Interval;

^a^ All listed variables (age group, sex, marital status, educational level, annual household income, region, area of residence, number of chronic physical diseases, presence of comorbid CMDs, and presence of sleep disturbances) were simultaneously entered into the negative binomial regression model to obtain adjusted RRs. Bold values represent statistically significant associations at P<0.05.

In terms of participants with 12-month MDs, higher baseline WHODAS-2.0 scores were associated with primary school education (vs. literate or below primary school: RR = 1.10; 95% CI: 1.00-1.20), residing in western regions (vs. Eastern: RR = 1.11; 95% CI: 1.01-1.22), comorbidity of CMDs (RR = 1.14; 95% CI: 1.06-1.23), three or more physical diseases (vs. none: RR = 1.23; 95% CI: 1.09-1.39), and sleep disturbances (RR = 1.21; 95% CI: 1.10-1.34).

Regarding individuals with 12-month ADs, higher baseline WHODAS-2.0 scores were associated with older age (≥70 years vs. 60–64 years: RR = 1.16; 95% CI: 1.08-1.25), comorbidity of CMDs (RR = 1.29; 95% CI: 1.21-1.38), physical diseases (1–2 physical diseases, RR = 1.19; 95% CI: 1.08-1.30; three or more physical diseases, RR = 1.35; 95% CI: 1.23-1.48), and sleep disturbances (RR = 1.14; 95% CI: 1.06-1.23).

Analyses were not performed for SUDs due to insufficient sample size to support stable statistical estimation.

### 3.4 Correlates of long-term disability

The results of the multivariate negative binomial regression on the correlates of five-year WHODAS-2.0 scores are shown in **Table 4**. Baseline WHODAS-2.0 score significantly predict follow-up WHODAS-2.0 score across all three group/subgroups (CMDs: RR 1.01, 95% CI 1.00-1.01, p=0.001; MDs: RR 1.01, 95% CI 1.00-1.01, p<0.001; ADs: RR 1.01, 95% CI 1.00-1.01, p<0.001). After adjustment for baseline disability, among individuals with 12-month CMDs, lower long-term WHODAS-2.0 scores were significantly associated with higher educational level (junior high school and above vs. literate or below primary school: RR = 0.85; 95% CI: 0.74-0.97) and urban residence (vs. rural: RR = 0.89, 95% CI: 0.79 -0.99).

**Table 4:** Demographic correlates of WHODAS 2.0 score at five-year follow-up among older adults regarding CMDs, MDs, ADs: multivariate negative binomial regression^a^.

Variables	Category	CMDs (n=134)		MDs (n=71)		ADs (n=86)	
		RR (95% CI)	*P*	RR (95% CI)	*P*	RR (95% CI)	*P*
Age	60-64	Ref.	-	Ref.	-	Ref.	-
	65-69	0.97 (0.86-1.09)	0.577	1.02 (0.85-1.23)	0.837	0.92 (0.80-1.07)	0.298
	≥70	1.09 (0.94-1.26)	0.258	1.08 (0.88-1.33)	0.454	1.09 (0.91-1.31)	0.360
Sex	Female	Ref.	-	Ref.	-	Ref.	-
	Male	0.91 (0.81-1.01)	0.079	0.85 (0.71-1.03)	0.098	0.93 (0.81-1.06)	0.270
Marital status	Married/Cohabitating	Ref.	-	Ref.	-	Ref.	-
	Separated/Widowed/Divorced/Never Married	1.00 (0.86-1.16)	0.987	0.88 (0.72-1.06)	0.177	0.94 (0.77-1.16)	0.580
Educational level	Literate or below primary school	Ref.	-	Ref.	-	Ref.	-
	Primary school	0.91 (0.80-1.03)	0.130	0.97 (0.80-1.17)	0.733	0.76 (0.63-0.92)	**0.005**
	Junior high school and above	0.85 (0.74-0.97)	**0.016**	0.77 (0.62-0.97)	**0.028**	0.89 (0.76-1.05)	0.157
Annual household income	<10500	Ref.	-	Ref.	-	Ref.	-
	10500-42499	0.90 (0.80-1.01)	0.079	0.78 (0.65-0.95)	**0.011**	0.88 (0.76-1.02)	0.083
	≥42500	0.89 (0.77-1.03)	0.133	0.72 (0.57-0.91)	**0.007**	0.86 (0.71-1.03)	0.107
Region	Eastern	Ref.	-	Ref.	-	Ref.	-
	Central	1.03 (0.91-1.16)	0.672	1.02 (0.85-1.21)	0.859	0.98 (0.84-1.14)	0.757
	Western	0.94 (0.82-1.07)	0.344	0.94 (0.78-1.13)	0.478	0.77 (0.65-0.92)	**0.004**
Area of residence	Rural	Ref.	-	Ref.	-	Ref.	-
	Urban	0.89 (0.79-0.99)	**0.035**	0.95 (0.79-1.15)	0.617	0.80 (0.69-0.92)	**0.001**
Baseline WHODAS 2.0 score		1.01 (1.00-1.01)	**0.001**	1.01 (1.00-1.01)	**<0.001**	1.01 (1.00-1.01)	**<0.001**

WHODAS, world health organization disability assessment schedule; CMDs, Common mental disorders; MDs, mood disorders; ADs, Anxiety disorders; RR, rate ratio; CI, Confidence Interval; ^a^Estimates models are based on the multivariate negative binomial models. All demographic variables (age group, sex, marital status, educational level, annual household income, region, and area of residence) were entered simultaneously with adjustment for baseline WHODAS 2.0 score to obtain adjusted RRs. Bold values represent statistically significant associations at P<0.05.

In terms of participants with 12-month MDs, lower long-term WHODAS-2.0 scores were significantly associated with higher annual household income (10,500-42,499 Yuan vs. <10500 Yuan: RR = 0.78, 95% CI: 0.65-0.95; ≥42500 Yuan vs. <10500 Yuan: RR = 0.72, 95% CI: 0.57–0.91) and higher educational level (junior high school and above vs. literate or below primary school: RR = 0.77, 95% CI: 0.62–0.97).

Regarding 12-month ADs, lower five-year WHODAS-2.0 scores were associated with primary school education (vs. literate or below primary school: RR = 0.76, 95% CI: 0.63–0.92), residence in the Western region (vs. Eastern: RR = 0.77, 95% CI: 0.65–0.92), and urban residence (vs. rural: RR = 0.80, 95% CI: 0.69–0.92).

Subgroup analyses were not performed for SUDs due to insufficient sample size to support stable statistical estimation.

The exploratory analyses yielded broadly consistent results across the three models, with detailed findings presented in **Supplementary Tables S5**-**S7** of the Supplementary Materials.

### 3.5 Sensitivity analyses

Sensitivity analyses comparing the prevalence of disability among participants with CMDs at baseline and five-year follow-up under unweighted and weighted approaches (**Supplementary Table S5**) showed that the weighted five-year prevalence was 19.3%, which was slightly lower than the unweighted estimate of 26.1%.

For baseline WHODAS-2.0 total score correlates (**Supplementary Table S9**), the overall patterns of associations were largely consistent between unweighted and weighted models. A few differences were observed: among participants with MDs, primary school education was significantly associated with higher WHODAS scores in the weighted analysis (RR = 1.10, 95% CI: 1.00–1.20) but not in the unweighted analysis; for area of residence, all comparisons were significant in the unweighted analysis (CMDs: RR = 0.93, 95% CI: 0.88-0.99; MDs: RR = 0.90, 95% CI: 0.83-0.98; ADs: RR = 0.93, 95% CI: 0.86-1.00), but none remained significant after weighting (all p>0.05).

As shown in **Supplementary Table S10**, for five-year follow-up WHODAS-2.0 total score correlates, the main findings were also consistent between unweighted and weighted analyses. Notable differences included higher educational level, which was non-significant unweighted but associated with lower follow-up WHODAS scores across all three categories after weighting. Among participants with MDs, higher household income was associated with lower WHODAS scores only in the weighted analysis. All other correlates were consistent across approaches.

## 4 Discussion

### 4.1 Main findings

Based on the nationally representative China Mental Health Survey, this five-year longitudinal study documents a high burden of disability among older adults with CMDs at baseline (57.1%) and persisted in 19.3% at follow-up, with clinical factors associated with baseline impairment and socioeconomic protective factors for long-term disability. Prior longitudinal studies, including the China Health and Retirement Longitudinal Study (CHARLS) and the Chinese Longitudinal Healthy Longevity Survey (CLHLS), have examined mental health and disability among older adults in China. Long-term monitoring of mental disorder burden in this populations underscores the importance of investigating persistent functional impairment related to mental health conditions (28). However, few studies have tracked disability trajectories and relevant associated factors specifically among community-dwelling older adults with CMDs diagnosed using standardized diagnostic instruments. The present study addresses this gap by examining disability status at baseline and after five years of follow-up, along with their respective correlates, in this population.

### 4.2 The prevalence of disability

The prevalence of substantial disability in older adults with 12-month any CMDs was 57.1% at baseline. MDs were linked to the highest disability prevalence, followed by ADs and SUDs. At the five-year follow-up, the overall prevalence was 19.3%, and the relative ordering of disability prevalence across diagnostic categories remained consistent with baseline.

Lower disability prevalence was observed among participants with CMDs overall and among the MD and AD subgroups at the five-year follow-up. However, this population-level pattern should not be simply interpreted as evidence of individual functional improvement. This pattern is partly consistent with a previous study showing that approximately 20% of older patients with depression experienced functional recovery two years later (29), potentially reflecting the natural waxing and waning course of MDs and ADs (30). Additionally, disability patterns may differ among various age groups. One study involving middle-aged and older adults with ADs reported a decreasing trend in disability prevalence with age among individuals over 60 years old, while an increasing trend was noted in the 45 to 59 years (31).

The weighted prevalence of substantial disability among participants with SUDs was 33.7% at baseline and 28.0% at the five-year follow-up. Older adults with SUDs face an heightened risk of developing neurotoxic effects and adverse events related to drug use (32). Furthermore, age-related alterations in the brain, along with changes in drug metabolism and pharmacokinetics, heighten the vulnerability of older adults to these adverse events (33, 34), which may contribute to persistent disability in this population.

A total of 19.3% of participants with CMDs experienced disability at the five-year follow-up. One plausible explanation for this persistence is recurrent episodes and prolonged symptom courses, which can lead to long-standing untreated symptoms and subsequent persistent functional impairment (35). The variation in disability status observed in our cohort may partly be explained by effective treatments or interventions, a mechanism supported by prior research, although we cannot directly test this hypothesis in the present study due to the lack of data on treatment uptake (36, 37). Prior research has shown that early prevention may be effective in offsetting functional impairments associated with mental disorders (31, 38), and appropriate interventions can lead to a substantial reduction in the associated burdens (39).

### 4.3 Correlates of disability severity at baseline

At baseline, physical comorbidity, comorbidity of multiple CMDs, and sleep disturbances were consistently associated with higher WHODAS-2.0 scores across all diagnostic groups, representing the most robust correlates of disability severity in this population.

The comorbidity of chronic physical diseases was an independent determinant of disability at baseline among participants with 12-month CMDs. This finding was reinforced by the results of an additional study indicating that chronic physical diseases were a contextual factor influencing disability in individuals suffering from mental disorders (40). Comorbidity of physical diseases may lead to poorer treatment outcomes and reduced adherence to treatment, which could further aggravate mental symptoms (41).

Additionally, the comorbidity of mental and physical disorders has been linked to increased severity of role dysfunction, resulting in more significant challenges in daily functioning (42). This finding implies that, in addition to treating older adults with CMDs, it is essential to manage physical diseases, which can effectively reduce disability promptly (43). Another finding from this research indicated that the comorbidity of CMDs was an independent determinant of disability at baseline. The presence of comorbid mental disorders frequently complicates clinical manifestations, resulting in a prolonged duration of illness and increased challenges in treatment (44), which can subsequently elevate the risk of disability.

An important factor associated with disability at baseline was sleep disturbance among participants with CMDs. Increased amyloid-beta protein (Aβ) levels in the cerebrospinal fluid occur under deprivation or low sleep efficiency, which may induce neurodegenerative changes. It is well established that neurodegeneration impairs the regeneration of muscle fibers, thereby diminishing the capacity to generate muscle strength, which is directly correlated with functional disability (45). Furthermore, older adults who experience poor sleep quality at night often engage in daytime napping, leading to difficulties in maintaining attention during the day, slower physical response times, and memory problems (46). Consequently, there are several behavioral and physiological pathways through which sleep disturbances may increase the risk of disability.

### 4.4 Correlates of long-term disability severity

The study found that participants from lower socioeconomic groups should be regarded as a key population for long-term disability among older adults with CMDs. The results of this study suggest that educational level predicts disability among older adults with CMDs five years later. Previous research has established that educational attainment is negatively associated with disability (47). Additionally, individuals with higher education tend to be more effective in managing illness and are increased willingness and capability to adapt their surroundings in order to handle disability (48). Among participants with MDs, higher annual household income was associated with lower disability prevalence at the five-year follow-up. As reported in earlier studies (39, 49), enhanced healthcare access and reduced financial strain constitute potential mechanisms that may account for this association.

Furthermore, participants living in urban areas with ADs experienced lower levels of disability five years later than their counterparts living in rural areas. Residing in urban environments offers relatively better living conditions and medical facilities, more robust social support systems, enhanced cultural and sports amenities, and increased opportunities for social engagement (50, 51), which may further reduce the likelihood of long-term disability. Therefore, in addition to providing timely treatment to alleviate the symptoms of disabled participants with ADs to alleviate their symptoms, there should be an ongoing focus on improving the functioning of participants living in rural areas.

## 5 Limitations

The results of this research need to be understood while considering certain limitations. Firstly, this study experienced a high attrition rate. To mitigate potential selection bias, we conducted a comprehensive attrition analysis comparing baseline characteristics between followed and lost participants and applied weighting in the analyses. Borderline differences in marital status and CMD diagnosis suggest selection bias, despite similar overall baseline disability and other key baseline characteristics between groups. Weighted–unweighted sensitivity analyses were broadly consistent, but under 77% attrition they cannot fully substitute for multiple imputation, particularly given that decedents were more disabled at baseline. Most importantly, among adults aged ≥60 years, selective mortality linked to disability cannot be ruled out and may bias trajectories. The observed weighted disability-prevalence drop (57.1% to 19.3%) may stem from selective survival rather than true functional improvement, precluding causal inferences. Accordingly, the high attrition rate may limit generalizability, and the results should be interpreted with caution. Secondly, both at baseline and five years later, disability was assessed based on the previous 30 days, while mental disorders were diagnosed based on symptoms experienced in the past year. In the case of episodic conditions, assessing disability based on the preceding month might not encompass the entire duration of the disorder, which could lead to an underestimation of the overall disability associated with CMDs. Assessment mode also changed between waves, from face-to-face CAPI at baseline to telephone CATI at follow-up, and some follow-up data were provided by proxy informants. Both mode and proxy effects may bias longitudinal comparisons, and because telephone interviewing may elicit lower disability reporting, the observed decline could be partly artefactual. Furthermore, disability was assessed only once at follow-up, precluding examination of continuous trajectories and limiting the ability to draw causal inferences. The follow-up interval varied slightly between participants, which may have introduced heterogeneity into the disability trajectories. Thirdly, we lacked data on treatment exposure, healthcare utilisation, relapse and illness course during follow-up, prohibiting empirical testing of potential explanatory mechanisms. Fourth, limited follow-up sample size may compromise the precision of regression estimates, particularly for disorder-specific subgroup analyses. Future research should verify vital status through official records, implement repeated outcome assessments and collect detailed clinical and service utilisation data over extended follow-up periods.

## 6 Conclusion

More than half (57.1%) of community-dwelling older adults with 12-month CMDs had substantial disability. Although the prevalence of disability was lower at the five-year follow-up, 19.3% of participants with CMDs who survived and who could be re-contacted remained disabled, with patterns varying by diagnostic category. Interventions should prioritise the management of physical and mental comorbidities and sleep disturbances to reduce disability. For long-term outcomes, targeted support for older adults in rural areas and those with lower educational attainment or household income may help reduce persistent disability across mental disorder types. This study provides new directions for improving treatment strategies and facilitates a more suitable distribution of limited healthcare resources to the older population affected.

## Data Availability

The original contributions presented in the study are included in the article/**Supplementary Material**, further inquiries can be directed to the corresponding author/s.

## References

[1] Cheng Y Fang Y Zheng J Guan S Wang M Hong W . The burden of depression, anxiety and schizophrenia among the older population in ageing and aged countries: an analysis of the Global Burden of Disease Study 2019. Gen Psychiatr. (2024) 371:e101078. doi: 10.1136/gpsych-2023-101078 38274290 PMC10806508

[2] Mental Health of Older Adults. Available online at: https://www.who.int/news-room/fact-sheets/detail/mental-health-of-older-adults (Accessed September 19, 2026).

[3] Guo C Li N Chen G Zheng X . Mental health service utilization and its associated social factors among elderly people with a mental disability in China: A national population-based survey. Scand J Public Health. (2019) 472:215–20. doi: 10.1177/1403494817722705 28784028

[4] Huang Y Wang Y Wang H Liu Z Yu X Yan J . Prevalence of mental disorders in China: a cross-sectional epidemiological study. Lancet Psychiatry. (2019) 63:211–24. doi: 10.1016/s2215-0366(18)30511-x 30792114

[5] Alsamiri YA Hussain MA Alsamani OA Al Bulayhi AA . Promoting mental health and wellbeing as means to prevent disability: a review. Front Public Health. (2024) 12:1425535. doi: 10.3389/fpubh.2024.1425535 39555044 PMC11563790

[6] Ramaprasad D Rao NS Kalyanasundaram S . Disability and quality of life among elderly persons with mental illness. Asian J Psychiatr. (2015) 18:31–6. doi: 10.1016/j.ajp.2015.10.007 26573889

[7] Nicholson PJ . Common mental disorders and work. Br Med Bull. (2018) 1261:113–21. doi: 10.1093/bmb/ldy014 29684103

[8] Garin N Olaya B Moneta MV Miret M Lobo A Ayuso-Mateos JL . Impact of multimorbidity on disability and quality of life in the Spanish older population. PloS One. (2014) 911:e111498. doi: 10.1371/journal.pone.0111498 25375890 PMC4222819

[9] Pan C Cao N Kelifa MO Luo S . Age and cohort trends in disability among Chinese older adults. Front Public Health. (2023) 11:998948. doi: 10.3389/fpubh.2023.998948 36969644 PMC10031081

[10] Kim T Park SY Oh IH . Health-related factors leading to disabilities in Korea: Survival analysis. Front Public Health. (2022) 10:1048044. doi: 10.3389/fpubh.2022.1048044 36620295 PMC9813747

[11] Amini F Khajevand Khoshli A Asadi J Bahar A Najafipour H Mirzazadeh A . Obesity mediates the effect of past and current mental health on diabetes treatment outcomes. Iran J Public Health. (2022) 5111:2608–18. doi: 10.18502/ijph.v51i11.11180 36561260 PMC9745415

[12] Morin RT Nelson C Bickford D Insel PS Mackin RS . Somatic and anxiety symptoms of depression are associated with disability in late life depression. Aging Ment Health. (2020) 248:1225–8. doi: 10.1080/13607863.2019.1597013 30945553 PMC9183787

[13] Tian G Li R Cui Y Zhou T Shi Y Yang W . Association between disability, social support and depressive symptoms in Chinese older adults: A national study. Front Public Health. (2022) 10:980465. doi: 10.3389/fpubh.2022.980465 36062100 PMC9437525

[14] Olaya B Moneta MV Koyanagi A Lara E Miret M Ayuso-Mateos JL . The joint association of depression and cognitive function with severe disability among community-dwelling older adults in Finland, Poland and Spain. Exp Gerontol. (2016) 76:39–45. doi: 10.1016/j.exger.2016.01.010 26805608

[15] Liu Z Li P Yin H Li M Yan J Ma C . Future trends in disability and its determinants among chinese community patients with anxiety disorders: evidence from a 5-year follow-up study. Front Psychiatry. (2021) 12:777236. doi: 10.3389/fpsyt.2021.777236 34955923 PMC8695844

[16] Zhang Y Li M Yin H Ma C Huang Z Yan Y . Long-term disability in common mental disorders in Chinese community: evidence from a five-year follow-up study. BMC Psychiatry. (2022) 221:727. doi: 10.1186/s12888-022-04382-4 36419029 PMC9682650

[17] Huang Y Liu Z Wang H Guan X Chen H Ma C . The China Mental Health Survey (CMHS): I. background, aims and measures. Soc Psychiatry Psychiatr Epidemiol. (2016) 5111:1559–69. doi: 10.1007/s00127-016-1270-z 27796403

[18] Liu Z Huang Y Lv P Zhang T Wang H Li Q . The China Mental Health Survey: II. Design and field procedures. Soc Psychiatry Psychiatr Epidemiol. (2016) 5111:1547–57. doi: 10.1007/s00127-016-1269-5 27803977

[19] Kessler RC Ustün TB . The world mental health (WMH) survey initiative version of the world health organization (WHO) composite international diagnostic interview (CIDI). Int J Methods Psychiatr Res. (2004) 132:93–121. doi: 10.1002/mpr.168 15297906 PMC6878592

[20] Kessler RC Birnbaum H Demler O Falloon IR Gagnon E Guyer M . The prevalence and correlates of nonaffective psychosis in the National Comorbidity Survey Replication (NCS-R). Biol Psychiatry. (2005) 588:668–76. doi: 10.1016/j.biopsych.2005.04.034 16023620 PMC2847859

[21] Kessler RC Merikangas KR . The National Comorbidity Survey Replication (NCS-R): background and aims. Int J Methods Psychiatr Res. (2004) 132:60–8. doi: 10.1002/mpr.166 15297904 PMC6878416

[22] Koopmans AB van Hoeken D Clarke DE Vinkers DJ van Harten PN Hoek HW . Proxy WHO disability assessment schedule 2.0 is clinically useful for assessing psychosocial functioning in severe mental illness. Front Psychiatry. (2020) 11:303. doi: 10.3389/fpsyt.2020.00303 32351419 PMC7174765

[23] Chiu TY Yen CF Chou CH Lin JD Hwang AW Liao HF . Development of traditional Chinese version of World Health Organization disability assessment schedule 2.0 36--item (WHODAS 2.0) in Taiwan: validity and reliability analyses. Res Dev Disabil. (2014) 3511:2812–20. doi: 10.1016/j.ridd.2014.07.009 25094056

[24] Von Korff M Crane PK Alonso J Vilagut G Angermeyer MC Bruffaerts R . Modified WHODAS-II provides valid measure of global disability but filter items increased skewness. J Clin Epidemiol. (2008) 6111:1132–43. doi: 10.1016/j.jclinepi.2007.12.009 18619808 PMC3277915

[25] Scott KM Von Korff M Alonso J Angermeyer MC Bromet E Fayyad J . Mental-physical co-morbidity and its relationship with disability: results from the World Mental Health Surveys. Psychol Med. (2009) 391:33–43. doi: 10.1017/s0033291708003188 18366819 PMC2637813

[26] Akram M Cerin E Lamb KE White SR . Modelling count, bounded and skewed continuous outcomes in physical activity research: beyond linear regression models. Int J Behav Nutr Phys Act. (2023) 201:57. doi: 10.1186/s12966-023-01460-y 37147664 PMC10163772

[27] Habtamu K Medhin G Selamu M Tirfessa K Hanlon C Fekadu A . Functional impairment among people diagnosed with depression in primary healthcare in rural Ethiopia: a comparative cross-sectional study. Int J Ment Health Syst. (2019) 13:50. doi: 10.1186/s13033-019-0305-8 31346349 PMC6635998

[28] Xiao Y Chen TT Zhang Z Liu L Du N . Changes in the disease burden of depressive disorders among middle-aged and older adults (Aged 45-89) in china over 30 years: insights from the global burden of disease study 2021. Int J Geriatr Psychiatry. (2025) 403:e70069. doi: 10.1002/gps.70069 40090859

[29] Wassink-Vossen S Collard RM Wardenaar KJ Verhaak PFM Rhebergen D Naarding P . Trajectories and determinants of functional limitations in late-life depression: A 2-year prospective cohort study. Eur Psychiatry. (2019) 62:90–6. doi: 10.1016/j.eurpsy.2019.09.003 31550583

[30] Wittchen HU Lieb R Pfister H Schuster P . The waxing and waning of mental disorders: evaluating the stability of syndromes of mental disorders in the population. Compr Psychiatry. (2000) 412 Suppl 1:122–32. doi: 10.1016/s0010-440x(00)80018-8 10746914

[31] Zhi Z Yan S Yijuan H Jiahuan Z Xiaohan J Dandan C . Trends in the disease burden of anxiety disorders in middle-aged and older adults in China. BMC Psychol. (2024) 121:83. doi: 10.1186/s40359-024-01575-2 38373999 PMC10877872

[32] Yarnell S Li L MacGrory B Trevisan L Kirwin P . Substance use disorders in later life: A review and synthesis of the literature of an emerging public health concern. Am J Geriatr Psychiatry. (2020) 282:226–36. doi: 10.1016/j.jagp.2019.06.005 31340887

[33] Wu LT Blazer DG . Illicit and nonmedical drug use among older adults: a review. J Aging Health. (2011) 233:481–504. doi: 10.1177/0898264310386224 21084724 PMC3097242

[34] Simoni-Wastila L Yang HK . Psychoactive drug abuse in older adults. Am J Geriatr Pharmacother. (2006) 44:380–94. doi: 10.1016/j.amjopharm.2006.10.002 17296542

[35] Gaspar FW Zaidel CS Dewa CS . Rates and predictors of recurrent work disability due to common mental health disorders in the United States. PloS One. (2018) 1310:e0205170. doi: 10.1371/journal.pone.0205170 30300392 PMC6177162

[36] Dong Y Zhang X Zhao R Cao L Kuang X Yao J . The effects of mind-body exercise on anxiety and depression in older adults: a systematic review and network meta-analysis. Front Psychiatry. (2024) 15:1305295. doi: 10.3389/fpsyt.2024.1305295 38384592 PMC10879425

[37] Yan Z Meng F He M Li Z . The efficacy of a transdiagnostic group cognitive behavioral intervention for Chinese elderly with emotional disorders: A one-year follow-up randomized clinical trial. Front Psychiatry. (2022) 13:1027994. doi: 10.3389/fpsyt.2022.1027994 36506431 PMC9732091

[38] Craske MG Stein MB . Anxiety. Lancet. (2016) 38810063:3048–59. doi: 10.1016/s0140-6736(16)30381-6 27349358

[39] Li N Du W Zhang L Chen G Zheng X . Prevalence and functions of mental disability caused by mood disorders in China: A national sample. J Affect Disord. (2015) 180:10–3. doi: 10.1016/j.jad.2015.03.016 25879719

[40] Hu Y Huang Y Wang L Liu Z Wang L Yan J . Disability and comorbidity of mood disorders and anxiety disorders with diabetes and hypertension: evidences from the china mental health survey and chronic disease surveillance in china. Front Psychiatry. (2022) 13:889823. doi: 10.3389/fpsyt.2022.889823 35669270 PMC9163306

[41] Berk M Köhler-Forsberg O Turner M Penninx B Wrobel A Firth J . Comorbidity between major depressive disorder and physical diseases: a comprehensive review of epidemiology, mechanisms and management. World Psychiatry. (2023) 223:366–87. doi: 10.1002/wps.21110 37713568 PMC10503929

[42] Sheridan PE Mair CA Quiñones AR . Associations between prevalent multimorbidity combinations and prospective disability and self-rated health among older adults in Europe. BMC Geriatr. (2019) 191:198. doi: 10.1186/s12877-019-1214-z 31351469 PMC6661084

[43] Quiñones AR Markwardt S Thielke S Rostant O Vásquez E Botoseneanu A . Prospective disability in different combinations of somatic and mental multimorbidity. J Gerontol A Biol Sci Med Sci. (2018) 732:204–10. doi: 10.1093/gerona/glx100 28541396 PMC6279134

[44] Yen S Stout R Hower H Killam MA Weinstock LM Topor DR . The influence of comorbid disorders on the episodicity of bipolar disorder in youth. Acta Psychiatr Scand. (2016) 1334:324–34. doi: 10.1111/acps.12514 26475572 PMC4801672

[45] Idalino SCC Cândido LM Wagner KJP de Souza Moreira B de Carvalho Bastone A Danielewicz AL . Association between sleep problems and functional disability in community-dwelling older adults. BMC Geriatr. (2024) 241:253. doi: 10.1186/s12877-024-04822-8 38481136 PMC10938775

[46] Berkley AS . Sleep, aging, and daily functioning. Nurs Clin North Am. (2021) 562:287–98. doi: 10.1016/j.cnur.2021.02.007 34023122

[47] Macinko J Vaz de Melo Mambrini J Bof de Andrade F Drumond Andrade FC Lazalde GE Lima-Costa MF . Life-course risk factors are associated with activity of daily living disability in older adults. Eur J Public Health. (2021) 313:520–7. doi: 10.1093/eurpub/ckaa156 33128061 PMC8485733

[48] Chen Y Sloan FA . Explaining disability trends in the U.S. Elderly and near-elderly population. Health Serv Res. (2015) 505:1528–49. doi: 10.1111/1475-6773.12284 25655273 PMC4600360

[49] Antunes A Frasquilho D Azeredo-Lopes S Silva M Cardoso G Caldas-de-Almeida JM . The effect of socioeconomic position in the experience of disability among people with mental disorders: findings from the World Mental Health Survey Initiative Portugal. Int J Equity Health. (2018) 171:113. doi: 10.1186/s12939-018-0821-1 30086758 PMC6081835

[50] Jin W Felix MS Paek SC Lamy FR . Social activities and depressive symptoms among elderly based on rural and urban differences in china: a national cross-sectional study. Psychol Res Behav Manag. (2024) 17:2243–54. doi: 10.2147/prbm.S460453 38855479 PMC11162184

[51] Liu Z Huang Y Chen H Ma C Zhang T . A cross-sectional survey of mental disability attributed to psychosis among persons aged 15 years and over in China. Chin Ment Health J. (2019) 3305:321–7. doi: 10.3969/j.issn.1000-6729.2019.05.001

