# Supplementary material for: Disability and its correlates among older adults with common mental disorders in China: a 5-year follow-up study
Source: Front Psychiatry. 2026 Oct 7;17:1907148. doi: 10.3389/fpsyt.2026.1907148 (PMC13683575; doi:10.3389/fpsyt.2026.1907148)
Supplement: Supplementary file 1 [file DataSheet1.pdf]

Table S1. Baseline characteristics of participants who were followed up versus those lost to follow-up (n=582)

Variables	Category	Lost to follow-up (n=448)		Followed up (n=134)		χ^2/Z	*P*
		Frequency n	Proportion (%)	Frequency n	Proportion (%)		
Age	60-64	196	43.8	70	52.2	3.0	0.223
	65-69	135	30.1	34	25.4		
	≥70	117	26.1	30	22.4		
Sex	Male	193	43.1	58	43.3	0.0	0.967
	Female	255	56.9	76	56.7		
Educational level	Literate/below primary school	234	52.2	61	45.5	2.4	0.294
	Primary school	125	27.9	39	29.1		
	Junior high school and above	89	19.9	34	25.4		
Marital status	Married/Cohabitating	325	72.5	109	81.3	4.2	**0.040**
	Separated/Widowed/Divorced/Never Married	123	27.5	25	18.7		
Annual household income	<10500	247	55.1	61	45.5	4.1	0.131
	10500-42499	122	27.2	42	31.3		
	≥42500	79	17.6	31	23.1		
Region	Eastern	134	29.9	37	27.6	2.0	0.371
	Central	174	38.8	61	45.5		
	Western	140	31.3	36	26.9		
Area of residence	Rural	264	58.9	74	55.2	0.6	0.446
	Urban	184	41.1	60	44.8		
Comorbidity of physical diseases	0	76	17.0	19	14.2	0.8	0.678
	1-2	179	40.0	58	43.3		
	≥3	193	43.0	57	42.5		
Diagnosis type	Mood disorders only	180	40.2	42	31.3	8.0	**0.047**

	Anxiety disorders only	167	37.3	57	42.5		
	Substance use disorders only	29	6.5	4	3.0		
	Comorbidity of CMDs	72	16.1	31	23.1		
Comorbidity of CMDs	No	376	83.9	103	76.9	3.5	0.060
	Yes	72	16.1	31	23.1		
Sleep disturbances	No	118	26.3	29	21.6	1.2	0.272
	Yes	330	73.7	105	78.4		
Baseline disability	No	212	47.3	63	47.0	0.0	0.950
	Yes	236	52.7	71	53.0		
Baseline WHODAS 2.0 total score [a]		55 (42-77)		53 (43-73)		-0.3	0.754

CMDs, Common mental disorders; WHODAS, World Health Organization Disability Assessment Schedule; [a]Baseline WHODAS total score is a continuous variable and is presented as median (interquartile range, IQR), compared using the Wilcoxon rank-sum test.

Table S2. Multivariable logistic regression analysis of baseline predictors of attrition (n=582)[a]

Variables	Category	OR (95% CI)	*P*
Age	60-64	Ref.	-
	65-69	0.72 (0.45-1.17)	0.281
	≥70	0.87 (0.52-1.47)	0.932
Sex	Male	Ref.	-
	Female	0.91 (0.59-1.40)	0.659
Educational level	Literate/below primary school	Ref.	-
	Primary school	1.18 (0.72-1.94)	0.994
	Junior high school and above	1.39 (0.80-2.43)	0.334
Marital status	Married/Cohabitating	Ref.	-
	Separated/Widowed/Divorced/Never Married	0.66 (0.39-1.12)	0.123
Annual household income	<10500	Ref.	-
	10500-42499	1.32 (0.83-2.10)	0.671
	≥42500	1.44 (0.83-2.50)	0.396
Region	Eastern	Ref.	-
	Central	1.28 (0.79-2.07)	0.219
	Western	0.98 (0.57-1.69)	0.548
Area of residence	Rural	Ref.	-
	Urban	1.07 (0.69-1.65)	0.778
Comorbidity of physical diseases	0	Ref.	-
	1-2	1.30 (0.71-2.40)	0.307
	≥3	1.09 (0.57-2.08)	0.839
Comorbidity of CMDs	No	Ref.	-
	Yes	1.56 (0.95-2.57)	0.080
Sleep disturbances	No	Ref.	-
	Yes	1.39 (0.84-2.31)	0.200
Baseline disability	No	Ref.	-
	Yes	0.98 (0.62-1.52)	0.913

CMDs, Common mental disorders; OR: Odds Ratio; CI, Confidence Interval; [a]All listed variables (age group, sex, educational level, marital status, annual household income, region, area of residence, number of comorbid physical diseases, presence of comorbid CMDs, sleep disturbances, and baseline disability) were simultaneously entered into the multivariable logistic regression model to obtain adjusted ORs.

Table S3. The weighted prevalence of disability among participants with CMDs for all diagnostic subgroups at baseline and five years later.

	2013			2018		
		Disability			Disability	
	N	n	Weighted % (95% CI)	N	n	Weighted % (95% CI)
**Mood disorders**	317	200	70.2 (62.1-78.4)	71	25	28.9 (10.3-47.4)
Depressive disorders	293	182	69.9 (60.9-78.8)	65	24	28.6 (8.6-48.7)
Major depressive disorder	237	163	74.9 (64.5-85.3)	57	22	25.4 (7.2-43.6)
Dysthymic disorder	106	76	86.4 (80.7-92.2)	22	9	20.3 (0.0-61.2) *
Depressive disorder not otherwise specified	48	15	46.2 (37.4-54.9)	6	2	51.9 (-) *
Bipolar disorders	19	13	70.5 (68.0-72.9)	6	1	32.1 (-) *
Bipolar disorders I	15	10	67.9 (60.8-74.9)	6	1	32.1 (-) *
Bipolar disorders II	2	2	100.0 (-) *	-	-	-
Bipolar disorder not otherwise specified	2	1	72.5 (-) *	-	-	-
Substance-induced mood disorder	6	6	100.0 (-) *	-	-	-
**Anxiety disorders**	315	166	56.4 (46.8-65.9)	86	21	14.9 (3.8-25.9)
Panic disorder	34	22	66.9 (60.9-72.8)	12	4	22.5 (0.0-100.0) *
Agoraphobia with/out panic disorder	18	14	87.5 (85.9-88.9)	8	4	45.8 (-) *
Specific phobia	164	83	54.6 (43.9-65.4)	44	9	11.9 (0.0-30.3) *
Social phobia	27	21	91.8 (87.8-95.9)	8	2	6.8 (2.2-11.5) *
Obsessive compulsive disorder	113	62	57.5 (41.9-72.9)	24	5	27.1 (0.0-69.1) *
Generalized anxiety disorder	19	11	60.9 (25.2-96.8) *	11	4	22.1 (0.0-100.0) *
Substance-induced anxiety disorder	14	13	92.7 (-) *	5	2	28.3 (-) *
**Substance use disorders**	57	20	33.7 (12.9-54.5)	9	2	28.0 (5.6-50.4) *
Alcohol use disorder	38	9	18.9 (1.4-36.4) *	6	1	36.0 (0.0-100.0) *
Drug use disorder	19	11	65.1 (-) *	3	1	13.1 (-) *
**Any common mental disorder**	582	307	57.1 (49.0-65.1)	134	35	19.3 (9.0-29.7)

CMDs, Common mental disorders; CI, Confidence Interval; *Estimates for these subgroups are uninterpretable due to the limited sample size

Table S4. Comparison of baseline and follow-up substantial- disability prevalence among participants with CMDs who completed the five- year follow- up assessment (n=134)[a]

	N	2013 Disability		2018 Disability		S	*P*
		n	Weighted % (95%CI)	n	Weighted % (95%CI)		
**Mood disorders**	71	46	64.6 (50.1-79.1)	25	28.9 (10.3-47.4)	13.4	**<0.001**
Depressive disorders	65	42	64.3 (48.6-80.1)	24	28.6 (8.6-48.7)	-	-
Major depressive disorder	57	40	75.9 (62.7-89.1)	22	25.4 (7.2-43.6)	-	-
Dysthymic disorder	22	17	88.7 (70.5-100.0)	9	20.3 (0.0-61.2)	-	-
Depressive disorder not otherwise specified	6	2	11.0 (-)*	2	51.9 (-) *	-	-
Bipolar disorders	6	4	67.8 (-)*	1	32.1 (-) *	-	-
Bipolar disorders I	6	4	67.8 (-)*	1	32.1 (-) *	-	-
**Anxiety disorders**	86	46	56.8 (41.5-72.1)	21	14.9 (3.8-25.9)	20.2	**<0.001**
Panic disorder	12	8	65.6 (22.9-100)	4	22.5 (0.0-100.0)	-	-
Agoraphobia with/out panic disorder	8	5	71.5 (-)*	4	45.8 (-) *	-	-
Specific phobia	44	23	66.2 (57.2-75.2)	9	11.9 (0.0-30.3)	-	-
Social phobia	8	6	85.5 (67.5-100)	2	6.8 (2.2-11.5)	-	-
Obsessive compulsive disorder	24	12	57.8 (3.1-100)	5	27.1 (0.0-69.1)	-	-
Generalized anxiety disorder	11	6	37.5 (0-100)	4	22.1 (0.0-100.0)	-	-
Substance-induced anxiety disorder	5	5	100 (-)*	2	28.3 (-) *	-	-
**Substance use disorders**	9	5	60.9 (0-100)	2	28.0 (5.6-50.4)	-	-
Alcohol use disorder	6	2	39.8 (0-100)	1	36.0 (0.0-100.0)	-	-
Drug use disorder	3	3	100 (-)*	1	13.1 (-) *	-	-
**Any CMDs**	134	71	51.7 (40.9-62.5)	35	19.3 (9.0-29.7)	24.9	**<0.001**

CMDs, Common mental disorders; CI, Confidence Interval; [a]McNemar's test was used to compare the prevalence of substantial disability between baseline and five-year follow-up among participants with complete data. Due to limited sample size, analyses were restricted to participants with any CMDs, mood disorders (MDs), and anxiety disorders (ADs); *The 95% CI could not be estimated due to the limited sample size.

Table S5. Exploratory analysis: correlates of WHODAS 2.0 score at five-year follow-up among older adults with CMDs across three models (n=134)

Variables	Category	Model 1[a]		Model 2[b]		Model 3[c]	
		RR (95% CI)	*P*	RR (95% CI)	*P*	RR (95% CI)	*P*
Age	60-64	Ref.	-	Ref.	-	Ref.	-
	65-69	0.92 (0.81-1.03)	0.148	0.97 (0.86-1.09)	0.577	0.98 (0.86-1.10)	0.700
	≥70	1.09 (0.95-1.26)	0.230	1.09 (0.94-1.26)	0.258	1.08 (0.93-1.24)	0.300
Sex	Female	Ref.	-	Ref.	-	Ref.	-
	Male	0.88 (0.79-0.98)	**0.022**	0.91 (0.81-1.01)	0.079	0.92 (0.82-1.02)	0.120
Marital status	Married/Cohabitating	Ref.	-	Ref.	-	Ref.	-
	Separated/Widowed/Divorced/Never Married	1.05 (0.91-1.21)	0.484	1.00 (0.86-1.16)	0.987	1.00 (0.87-1.16)	0.993
Educational level	Literate or below primary school	Ref.	-	Ref.	-	Ref.	-
	Primary school	0.92 (0.81-1.04)	0.190	0.91 (0.80-1.03)	0.130	0.91 (0.80-1.04)	0.152
	Junior high school and above	0.81 (0.72-0.92)	**0.001**	0.85 (0.74-0.97)	**0.016**	0.83 (0.72-0.96)*	**0.011**
Annual household income	<10500	Ref.	-	Ref.	-	Ref.	-
	10500-42499	0.91 (0.81-1.03)	0.124	0.90 (0.80-1.01)	0.079	0.90 (0.80-1.02)	0.098
	≥42500	0.82 (0.72-0.94)	**0.004**	0.89 (0.77-1.03)	0.133	0.90 (0.78-1.05)	0.176
Region	Eastern	Ref.	-	Ref.	-	Ref.	-
	Central	0.96 (0.85-1.08)	0.458	1.03 (0.91-1.16)	0.672	1.03 (0.91-1.16)	0.663
	Western	0.94 (0.82-1.08)	0.359	0.94 (0.82-1.07)	0.344	0.93 (0.81-1.06)	0.289
Area of residence	Rural	Ref.	-	Ref.	-	Ref.	-
	Urban	0.84 (0.76-0.93)	**0.001**	0.89 (0.79-0.99)	**0.035**	0.89 (0.79-1.00)	0.053
Comorbidity of physical diseases at baseline	0	Ref.	-	-	-	Ref.	-
	1-2	0.97 (0.82-1.16)	0.744	-	-	0.93 (0.78-1.11)	0.413
	≥3	1.10 (0.92-1.31)	0.315	-	-	1.02 (0.85-1.24)	0.804
Comorbidity of CMDs at baseline	No	Ref.	-	-	-	Ref.	-
	Yes	1.06 (0.94-1.21)	0.355	-	-	1.02 (0.90-1.16)	0.709
Sleep disturbances at baseline	No	Ref.	-	-	-	Ref.	-
	Yes	1.10 (0.96-1.26)	0.158	-	-	1.02 (0.89-1.18)	0.742

WHODAS, World Health Organization Disability Assessment Schedule; CMDs, Common mental disorders; RR, Risk Ratio; CI, Confidence Interval; [a]Model 1: each variable was entered separately into the negative binomial regression model alongside baseline WHODAS 2.0 score. [b]Model 2: all demographic variables (age group, sex, marital status, educational level, annual household income, region, and area of residence) were entered simultaneously with adjustment for baseline WHODAS 2.0 score; [c]Model 3: all listed variables (age group, sex, marital status, educational level, annual household income, region, area of residence, number of chronic physical diseases, presence of comorbid CMDs, presence of sleep disturbances) and baseline WHODAS 2.0 score were simultaneously entered into the model to obtain adjusted RRs.

Table S6. Exploratory analysis: correlates of WHODAS 2.0 score at five-year follow-up among older adults with MDs across three models (n=71)

Variables	Category	Model 1[a]		Model 2[b]		Model 3[c]	
		RR (95% CI)	*P*	RR (95% CI)	*P*	RR (95% CI)	*P*
Age	60-64	Ref.	-	Ref.	-	Ref.	-
	65-69	0.90 (0.75-1.09)	0.286	1.02 (0.85-1.23)	0.837	1.01 (0.81-1.26)	0.922
	≥70	1.04 (0.85-1.28)	0.681	1.08 (0.88-1.33)	0.454	1.00 (0.82-1.23)	0.966
Sex	Female	Ref.	-	Ref.	-	Ref.	-
	Male	0.82 (0.68-0.98)	**0.028**	0.85 (0.71-1.03)	0.098	0.85 (0.70-1.03)	0.090
Marital status	Married/Cohabitating	Ref.	-	Ref.	-	Ref.	-
	Separated/Widowed/Divorced/Never Married	0.94 (0.77-1.14)	0.514	0.88 (0.72-1.06)	0.177	0.87 (0.71-1.06)	0.173
Educational level	Literate or below primary school	Ref.	-	Ref.	-	Ref.	-
	Primary school	0.89 (0.75-1.06)	0.194	0.97 (0.80-1.17)	0.733	0.98 (0.80-1.20)	0.865
	Junior high school and above	0.70 (0.58-0.85)	**<0.001**	0.77 (0.62-0.97)	**0.028**	0.84 (0.66-1.06)	0.142
Annual household income	<10500	Ref.	-	Ref.	-	Ref.	-
	10500-42499	0.81 (0.67-0.98)	**0.028**	0.78 (0.65-0.95)	**0.011**	0.81 (0.66-0.98)	**0.034**
	≥42500	0.71 (0.59-0.86)	**<0.001**	0.72 (0.57-0.91)	**0.007**	0.73 (0.56-0.94)	**0.015**
Region	Eastern	Ref.	-	Ref.	-	Ref.	-
	Central	0.93 (0.78-1.12)	0.441	1.02 (0.85-1.21)	0.859	0.98 (0.82-1.16)	0.793
	Western	0.92 (0.75-1.13)	0.424	0.94 (0.78-1.13)	0.478	0.81 (0.66-0.99)	**0.040**
Area of residence	Rural	Ref.	-	Ref.	-	Ref.	-
	Urban	0.79 (0.67-0.92)	**0.003**	0.95 (0.79-1.15)	0.617	0.94 (0.76-1.16)	0.549
Comorbidity of physical diseases at baseline	0	Ref.	-	-	-	Ref.	-
	1-2	0.96 (0.71-1.30)	0.795	-	-	1.00 (0.76-1.31)	0.999
	≥3	1.10 (0.81-1.51)	0.530	-	-	1.17 (0.88-1.55)	0.274
Comorbidity of CMDs at baseline	No	Ref.	-	-	-	Ref.	-
	Yes	0.98 (0.82-1.16)	0.794	-	-	0.90 (0.76-1.07)	0.238
Sleep disturbances at baseline	No	Ref.	-	-	-	Ref.	-
	Yes	1.15 (0.92-1.44)	0.213	-	-	1.04 (0.80-1.33)	0.790

WHODAS, World Health Organization Disability Assessment Schedule; CMDs, Common mental disorders; MDs, Mood disorders; RR, Risk Ratio; CI, Confidence Interval; [a]Model 1: each variable was entered separately into the negative binomial regression model alongside baseline WHODAS 2.0 score. [b]Model 2: all demographic variables (age group, sex, marital status, educational level, annual household income, region, and area of residence) were entered simultaneously with adjustment for baseline WHODAS 2.0 score; [c]Model 3: all listed variables (age group, sex, marital status, educational level, annual household income, region, area of residence, number of chronic physical diseases, presence of comorbid CMDs, presence of sleep disturbances) and baseline WHODAS 2.0 score were simultaneously entered into the model to obtain adjusted RRs.

Table S7 Exploratory analysis: correlates of WHODAS 2.0 score at five-year follow-up among older adults with ADs across three models (n=86)

Variables	Category	Model 1[a]		Model 2[b]		Model 3[c]	
		RR (95% CI)	*P*	RR (95% CI)	*P*	RR (95% CI)	*P*
Age	60-64	Ref.	-	Ref.	-	Ref.	-
	65-69	0.85 (0.73-0.99)	**0.032**	0.92 (0.80-1.07)	0.298	0.98 (0.82-1.17)	0.827
	≥70	1.01 (0.83-1.21)	0.949	1.09 (0.91-1.31)	0.360	1.06 (0.88-1.26)	0.546
Sex	Female	Ref.	-	Ref.	-	Ref.	-
	Male	0.92 (0.80-1.06)	0.240	0.93 (0.81-1.06)	0.270	0.98 (0.84-1.13)	0.769
Marital status	Married/Cohabitating	Ref.	-	Ref.	-	Ref.	-
	Separated/Widowed/Divorced/Never Married	1.01 (0.84-1.23)	0.880	0.94 (0.77-1.16)	0.580	1.01 (0.81-1.26)	0.927
Educational level	Literate or below primary school	Ref.	-	Ref.	-	Ref.	-
	Primary school	0.88 (0.74-1.04)	0.130	0.76 (0.63-0.92)	**0.005**	0.78 (0.65-0.95)	**0.012**
	Junior high school and above	0.82 (0.70-0.96)	**0.011**	0.89 (0.76-1.05)	0.157	0.85 (0.71-1.01)	**0.071**
Annual household income	<10500	Ref.	-	Ref.	-	Ref.	-
	10500-42499	0.93 (0.80-1.08)	0.333	0.88 (0.76-1.02)	0.083	0.92 (0.79-1.07)	0.302
	≥42500	0.82 (0.69-0.97)	**0.020**	0.86 (0.71-1.03)	0.107	0.87 (0.71-1.06)	0.176
Region	Eastern	Ref.	-	Ref.	-	Ref.	-
	Central	0.91 (0.79-1.05)	0.194	0.98 (0.84-1.14)	0.757	0.98 (0.83-1.16)	0.829
	Western	0.84 (0.70-1.00)	**0.048**	0.77 (0.65-0.92)	**0.004**	0.75 (0.62-0.9)	**0.002**
Area of residence	Rural	Ref.	-	Ref.	-	Ref.	-
	Urban	0.79 (0.69-0.90)	**<0.001**	0.80 (0.69-0.92)	**0.001**	0.79 (0.68-0.92)	**0.002**
Comorbidity of physical diseases at baseline	0	Ref.	-	-	-	Ref.	-
	1-2	0.91 (0.72-1.14)	0.194	-	-	0.87 (0.68-1.11)	0.259
	≥3	1.05 (0.84-1.32)	**0.048**	-	-	1.00 (0.79-1.28)	0.985
Comorbidity of CMDs at baseline	No	Ref.	-	-	-	Ref.	-
	Yes	1.17 (1.01-1.36)	**0.033**	-	-	1.06 (0.91-1.24)	0.443
Sleep disturbances at baseline	No	Ref.	-	-	-	Ref.	-
	Yes	1.09 (0.91-1.31)	0.338	-	-	1.04 (0.85-1.28)	0.705

WHODAS, World Health Organization Disability Assessment Schedule; CMDs, Common mental disorders; ADs, Anxiety disorders; RR, Risk Ratio; CI, Confidence Interval; [a]Model 1: each variable was entered separately into the negative binomial regression model alongside baseline WHODAS 2.0 score. [b]Model 2: all demographic variables (age group, sex, marital status, educational level, annual household income, region, and area of residence) were entered simultaneously with adjustment for baseline WHODAS 2.0 score; [c]Model 3: all listed variables (age group, sex, marital status, educational level, annual household income, region, area of residence, number of chronic physical diseases, presence of comorbid CMDs, presence of sleep disturbances) and baseline WHODAS 2.0 score were simultaneously entered into the model to obtain adjusted RRs.

Table S8 Sensitivity analysis: prevalence of disability among participants with CMDs at baseline and five years later under unweighted and weighted analytical approaches

	2013				2018			
	N	Disability			N	Disability		
		n	Unweighted %	Weighted %		n	Unweighted %	Weighted %
**Mood disorders**	317	200	63.1	70.2	71	25	35.2	28.9
Depressive disorders	293	182	62.1	69.9	65	24	36.9	28.6
Major depressive disorder	237	163	68.8	74.9	57	22	38.6	25.4
Dysthymic disorder	106	76	71.7	86.4	22	9	40.9	20.3
Depressive disorder not otherwise specified	48	15	31.3	46.2	6	2	33.3	51.9
Bipolar disorders	19	13	68.4	70.5	6	1	16.7	32.1
Bipolar disorders I	15	10	66.7	67.9	6	1	16.7	32.1
Bipolar disorders II	2	2	100.0	100.0	-	-	-	-
Bipolar disorder not otherwise specified	2	1	50.0	72.5	-	-	-	-
Substance-induced mood disorder	6	6	100.0	100.0	-	-	-	-
**Anxiety disorders**	315	166	52.7	56.4	86	21	24.4	14.9
Panic disorder	34	22	64.7	66.9	12	4	33.3	22.5
Agoraphobia with/out panic disorder	18	14	77.8	87.5	8	4	50.0	45.8
Specific phobia	164	83	50.6	54.6	44	9	20.5	11.9
Social phobia	27	21	77.8	91.8	8	2	25.0	6.8
Obsessive compulsive disorder	113	62	54.9	57.5	24	5	20.8	27.1
Generalized anxiety disorder	19	11	57.9	60.9	11	4	36.4	22.1
Substance-induced anxiety disorder	14	13	92.9	92.7	5	2	40.0	28.3
**Substance use disorders**	57	20	35.1	33.7	9	2	22.2	28.0
Alcohol use disorder	38	9	23.7	18.9	6	1	16.7	36.0
Drug use disorder	19	11	57.9	65.1	3	1	33.3	13.1
**Any CMDs**	582	307	52.8	57.1	134	35	26.1	19.3

CMDs, Common mental disorders.

Table S9 Sensitivity analysis: correlates of baseline WHODAS 2.0 score under unweighted and weighted negative binomial regression models[a]

Variables	Category	CMDs (n=582)				MDs (n=317)				ADs (n=315)			
		Unweighted		Weighted		Unweighted		Weighted		Unweighted		Weighted	
		RR (95% CI)	*P*	RR (95% CI)	*P*	RR (95% CI)	*P*	RR (95% CI)	*P*	RR (95% CI)	*P*	RR (95% CI)	*P*
Age	60-64	Ref.	-	Ref.	-	Ref.	-	Ref.	-	Ref.	-	Ref.	-
	65-69	1.00 (0.94-1.06)	0.946	1.01 (0.95-1.07)	0.786	0.97 (0.89-1.07)	0.561	0.92 (0.84-1.01)	0.076	1.01 (0.93-1.09)	0.873	1.05 (0.98-1.14)	0.187
	≥70	1.08 (1.01-1.16)	**0.026**	1.12 (1.06-1.19)	**<0.001**	1.02 (0.92-1.13)	0.691	1.03 (0.95-1.13)	0.487	1.14 (1.05-1.25)	**0.003**	1.16 (1.08-1.25)	**<0.001**
Sex	Female	Ref.	-	Ref.	-	Ref.	-	Ref.	-	Ref.	-	Ref.	-
	Male	0.99 (0.94-1.05)	0.819	0.97 (0.92-1.02)	0.196	1.03 (0.95-1.12)	0.508	0.99 (0.92-1.08)	0.896	1.07 (0.99-1.15)	0.087	1.04 (0.98-1.11)	0.221
Marital status	Married/Cohabitating	Ref.	-	Ref.	-	Ref.	-	Ref.	-	Ref.	-	Ref.	-
	Separated/Widowed/ Divorced/Never Married	1.02 (0.96-1.09)	0.536	0.98 (0.93-1.05)	0.596	1.03 (0.95-1.12)	0.493	1.01 (0.93-1.10)	0.754	1.06 (0.97-1.16)	0.170	1.05 (0.98-1.13)	0.192
Educational level	Literate/below primary school	Ref.	-	Ref.	-	Ref.	-	Ref.	-	Ref.	-	Ref.	-
	Primary school	1.02 (0.95-1.09)	0.565	1.03 (0.97-1.10)	0.294	1.05 (0.96-1.16)	0.279	1.10 (1.00-1.20)	**0.039**	0.96 (0.88-1.05)	0.396	0.96 (0.89-1.04)	0.321
	Junior high school and above	0.96 (0.89-1.04)	0.342	0.99 (0.92-1.06)	0.699	0.96 (0.85-1.07)	0.421	1.03 (0.93-1.14)	0.616	0.94 (0.86-1.04)	0.254	0.96 (0.88-1.04)	0.274
Annual household income	<10500	Ref.	-	Ref.	-	Ref.	-	Ref.	-	Ref.	-	Ref.	-
	10500-42499	0.97 (0.91-1.03)	0.303	0.98 (0.92-1.03)	0.436	0.96 (0.88-1.05)	0.365	0.96 (0.89-1.05)	0.374	0.98 (0.91-1.06)	0.627	1.00 (0.94-1.07)	0.941
	≥42500	1.00 (0.93-1.08)	0.908	0.97 (0.90-1.04)	0.340	1.05 (0.94-1.18)	0.369	0.97 (0.87-1.09)	0.644	1.03 (0.94-1.13)	0.581	1.02 (0.94-1.11)	0.587

Region	Eastern	Ref.	-	Ref.	-	Ref.	-	Ref.	-	Ref.	-	Ref.	-
	Central	1.02	0.646	1.06	0.070	1.04	0.422	1.09	0.074	0.98	0.622	1.02	0.537
		(0.95-1.08)		(1.00-1.12)		(0.94-1.15)		(0.99-1.19)		(0.90-1.06)		(0.95-1.10)	
	Western	1.09	**0.017**	1.09	**0.009**	1.11	**0.042**	1.11	**0.024**	1.04	0.440	1.06	0.110
		(1.02-1.17)		(1.02-1.16)		(1.00-1.23)		(1.01-1.22)		(0.95-1.13)		(0.99-1.15)	
Area of	Rural	Ref.	-	Ref.	-	Ref.	-	Ref.	-	Ref.	-	Ref.	-
residence	Urban	0.93	**0.022**	0.97	0.255	0.90	**0.020**	0.98	0.641	0.93	**0.037**	0.96	0.228
		(0.88-0.99)		(0.92-1.02)		(0.83-0.98)		(0.90-1.06)		(0.86-1.00)		(0.90-1.02)	
Comorbidity	0	Ref.	-	Ref.	-	Ref.	-	Ref.	-	Ref.	-	Ref.	-
of physical	1-2	1.12	**0.006**	1.10	**0.016**	1.11	0.119	1.04	0.492	1.17	**0.003**	1.19	**<0.001**
diseases		(1.03-1.21)		(1.02-1.18)		(0.97-1.26)		(0.92-1.18)		(1.06-1.29)		(1.08-1.30)	
	≥3	1.35	**<0.001**	1.31	**<0.001**	1.30	**<0.001**	1.23	**<0.001**	1.38	**<0.001**	1.35	**<0.001**
		(1.24-1.46)		(1.22-1.41)		(1.15-1.48)		(1.09-1.39)		(1.24-1.53)		(1.23-1.48)	
Comorbidity	No	Ref.	-	Ref.	-	Ref.	-	Ref.	-	Ref.	-	Ref.	-
of CMDs	Yes	1.18	**<0.001**	1.21	**<0.001**	1.11	**0.011**	1.14	**<0.001**	1.27	**<0.001**	1.29	**<0.001**
		(1.10-1.27)		(1.14-1.29)		(1.02-1.21)		(1.06-1.23)		(1.17-1.37)		(1.21-1.38)	
Sleep	No	Ref.	-	Ref.	-	Ref.	-	Ref.	-	Ref.	-	Ref.	-
disturbances	Yes	1.19	**<0.001**	1.17	**<0.001**	1.24	**<0.001**	1.21	**<0.001**	1.15	**0.001**	1.14	**<0.001**
		(1.11-1.27)		(1.10-1.24)		(1.12-1.38)		(1.10-1.34)		(1.06-1.26)		(1.06-1.23)	

WHODAS, World Health Organization Disability Assessment Schedule; CMDs, Common mental disorders; MDs, Mood disorders; ADs, Anxiety disorders; RR, Risk Ratio; CI, Confidence Interval; [a]All listed variables (age group, sex, marital status, educational level, annual household income, region, area of residence, number of chronic physical diseases, presence of comorbid CMDs, and presence of sleep disturbances) were simultaneously entered into the negative binomial regression model to obtain adjusted RRs.

Table S10. Sensitivity analysis: correlates of WHODAS 2.0 score at five-year follow-up under unweighted and weighted negative binomial regression models[a]

Variables	Category	CMDs (n=134)				MDs (n=71)				ADs (n=86)			
		Unweighted		Weighted		Unweighted		Weighted		Unweighted		Weighted	
		RR (95% CI)	*P*	RR (95% CI)	*P*	RR (95% CI)	*P*	RR (95% CI)	*P*	RR (95% CI)	*P*	RR (95% CI)	*P*
Age	60-64	Ref.	-	Ref.	-	Ref.	-	Ref.	-	Ref.	-	Ref.	-
	65-69	1.02 (0.89-1.17)	0.807	0.97 (0.86-1.09)	0.577	0.97 (0.79-1.19)	0.774	1.02 (0.85-1.23)	0.837	1.08 (0.91-1.28)	0.388	0.92 (0.80-1.07)	0.298
	≥70	1.13 (0.97-1.32)	0.111	1.09 (0.94-1.26)	0.258	1.18 (0.95-1.46)	0.143	1.08 (0.88-1.33)	0.454	1.03 (0.86-1.25)	0.734	1.09 (0.91-1.31)	0.360
Sex	Female	Ref.	-	Ref.	-	Ref.	-	Ref.	-	Ref.	-	Ref.	-
	Male	0.92 (0.81-1.03)	0.158	0.91 (0.81-1.01)	0.079	0.91 (0.74-1.11)	0.356	0.85 (0.71-1.03)	0.098	0.96 (0.83-1.11)	0.611	0.93 (0.81-1.06)	0.270
Marital status	Married/Cohabitating	Ref.	-	Ref.	-	Ref.	-	Ref.	-	Ref.	-	Ref.	-
	Separated/Widowed/ Divorced/Never Married	1.10 (0.94-1.29)	0.232	1.00 (0.86-1.16)	0.987	0.95 (0.77-1.16)	0.607	0.88 (0.72-1.06)	0.177	1.17 (0.92-1.48)	0.191	0.94 (0.77-1.16)	0.580
Educational level	Literate/below primary school	Ref.	-	Ref.	-	Ref.	-	Ref.	-	Ref.	-	Ref.	-
	Primary school	0.89 (0.78-1.03)	0.114	0.91 (0.80-1.03)	0.130	0.93 (0.75-1.15)	0.514	0.97 (0.80-1.17)	0.733	0.83 (0.68-1.02)	0.076	0.76 (0.63-0.92)	**0.005**
	Junior high school and above	0.89 (0.75-1.05)	0.179	0.85 (0.74-0.97)	**0.016**	0.89 (0.68-1.17)	0.413	0.77 (0.62-0.97)	**0.028**	0.90 (0.74-1.09)	0.276	0.89 (0.76-1.05)	0.157
Annual household income	<10500	Ref.	-	Ref.	-	Ref.	-	Ref.	-	Ref.	-	Ref.	-
	10500-42499	0.92 (0.81-1.05)	0.210	0.90 (0.80-1.01)	0.079	0.84 (0.68-1.03)	0.089	0.78 (0.65-0.95)	**0.011**	0.93 (0.80-1.09)	0.388	0.88 (0.76-1.02)	0.083
	≥42500	0.95 (0.80-1.13)	0.558	0.89 (0.77-1.03)	0.133	0.82 (0.63-1.07)	0.149	0.72 (0.57-0.91)	**0.007**	0.94 (0.74-1.18)	0.573	0.86 (0.71-1.03)	0.107

Region	Eastern	Ref.	-	Ref.	-	Ref.	-	Ref.	-	Ref.	-	Ref.	-
	Central	1.00 (0.87-1.15)	0.980	1.03 (0.91-1.16)	0.672	0.90 (0.74-1.10)	0.303	1.02 (0.85-1.21)	0.859	0.95 (0.80-1.13)	0.563	0.98 (0.84-1.14)	0.757
	Western	0.94 (0.81-1.09)	0.419	0.94 (0.82-1.07)	0.344	0.94 (0.75-1.16)	0.544	0.94 (0.78-1.13)	0.478	0.77 (0.64-0.93)	**0.007**	0.77 (0.65-0.92)	**0.004**
Area of residence	Rural	Ref.	-	Ref.	-	Ref.	-	Ref.	-	Ref.	-	Ref.	-
	Urban	0.87 (0.77-0.99)	**0.028**	0.89 (0.79-0.99)	**0.035**	0.97 (0.80-1.18)	0.770	0.95 (0.79-1.15)	0.617	0.76 (0.65-0.89)	**<0.001**	0.80 (0.69-0.92)	**0.001**

WHODAS, World Health Organization Disability Assessment Schedule; CMDs, Common mental disorders; MDs, Mood disorders; ADs, Anxiety disorders; RR, Risk Ratio; CI, Confidence Interval; [a]All listed variables (age group, sex, marital status, educational level, annual household income, region, and area of residence) were simultaneously entered into the negative binomial regression model to obtain adjusted RRs.
